# Granzyme B-inhibitor serpina3n induces neuroprotection in vitro and in vivo

**DOI:** 10.1186/s12974-015-0376-7

**Published:** 2015-09-04

**Authors:** Yohannes Haile, Katia Carmine-Simmen, Camille Olechowski, Bradley Kerr, R. Chris Bleackley, Fabrizio Giuliani

**Affiliations:** Department of Medicine, Division of Neurology, University of Alberta, 4C Kaye Edmonton Clinic, Edmonton, Alberta T6G 1Z1 Canada; Department of Biochemistry, University of Alberta, Edmonton, Canada; Department of Anesthesiology and Pain Medicine, University of Alberta, Edmonton, Canada; Department of Pharmacology, University of Alberta, Edmonton, Canada; Centre for Neuroscience, University of Alberta, Edmonton, Canada

**Keywords:** Multiple sclerosis, EAE, serpina3n, Granzyme B, Neurodegeneration

## Abstract

**Background:**

Multiple sclerosis (MS) is an autoimmune inflammatory and neurodegenerative disease of the central nervous system (CNS). It is widely accepted that inflammatory cells play major roles in the pathogenesis of MS, possibly through the use of serine protease granzyme B (GrB) secreted from the granules of cytotoxic T cells. We have previously identified GrB as a mediator of axonal injury and neuronal death. In this study, our goal was to evaluate the effect of GrB inhibition in the human system in vitro, and in vivo in EAE using the newly isolated GrB-inhibitor serpina3n.

**Methods:**

We used a well-established in vitro model of neuroinflammation characterized by a co-culture system between human fetal neurons and lymphocytes. In vivo, we induced EAE in 10- to 12-week-old female C57/BL6 mice and treated them intravenously with serpina3n.

**Results:**

In the in vitro co-culture system, pre-treatment of lymphocytes with serpina3n prevented neuronal killing and cleavage of the cytoskeletal protein alpha-tubulin, a known substrate for GrB. Moreover, in EAE, 50 μg serpina3n substantially reduced the severity of the disease. This dose was administered intravenously twice at days 7 and 20 post EAE induction. serpina3n treatment reduced axonal and neuronal injury compared to the vehicle-treated control group and maintained the integrity of myelin. Interestingly, serpina3n treatment did not seem to reduce the infiltration of immune cells (CD4^+^ and CD8^+^ T cells) into the CNS.

**Conclusion:**

Our data suggest further studies on serpina3n as a potentially novel therapeutic strategy for the treatment of inflammatory-mediated neurodegenerative diseases such as MS.

**Electronic supplementary material:**

The online version of this article (doi:10.1186/s12974-015-0376-7) contains supplementary material, which is available to authorized users.

## Introduction

Multiple sclerosis (MS) is an inflammatory, neurodegenerative and demyelinating autoimmune disease of the central nervous system (CNS) and is the most common cause of non-traumatic chronic neurologic disability [1–3]. Neurodegeneration is the main cause of disease progression in MS patients [4]; however, the detailed mechanisms that mediate this neuronal injury/death are not yet established [5]. A known fact is that the level of lymphocyte infiltration into the CNS is well controlled under normal conditions; in inflammatory disease states, however, unlimited number of T-lymphocytes cross the blood brain barrier (BBB) and enter the CNS compartment [6, 7]. These infiltrating T-lymphocytes are abundantly found within MS lesions [8, 9] and seem to be implicated in axonal pathology and neuronal death [10]. Indeed, we have previously shown that anti-CD3 activated T cells induce severe neurotoxic effect in both allogeneic and syngeneic systems in vitro [11]. However, the mechanisms of T cell-mediated neuronal injury/death have not been adequately explored.

Granzyme B (GrB) is a 32 kDa serine protease released from the granules of cytotoxic T cells [12] or secreted by T-lymphocytes when activated in vitro [13, 14]. There are five types of granzymes in humans and about 11 in mice [15, 16], but GrB is the most potent in both species and well characterized [17]. It has been observed that GrB expressing cytotoxic T cells are often found in close proximity of oligodendrocytes or demyelinating axons in acute MS lesions [18] and are associated with neuronal loss [19]. When these cytotoxic T cells come into contact with a target cell, they deliver a “lethal hit” of cytolytic molecules mainly constituted by perforin and GrB [20–22]. These molecules induce target cell death by disrupting a variety of intra/extracellular protein substrates [23–25]. We have previously reported that active MS lesions express high level of GrB. In vitro, granule-purified human GrB induces severe neurotoxic effects on human neurons to the same extent as activated T cells do. This was further confirmed by the observation that T cells isolated from GrB knockout BL6 mice were not able to kill neurons derived from syngeneic naïve mice [14]. In addition, T cell-mediated neurotoxicity was reduced by decreasing the levels of GrB within T cells [26]. We showed that purified human GrB internalizes into neuronal cells possibly through M6P receptor and induces neurotoxicity independent of perforin and in the absence of lytic agent in the cytoplasm [14]. All of these reports highlight GrB as a major player in T cell-mediated neuronal injury/death in the context of inflammatory-mediated neurodegenerative diseases such as MS and makes GrB a potentially attractive therapeutic target for these diseases.

In MS, the currently available disease-modifying treatments such as interferon β and glatiramer acetate reduce disease activity by ~30 % in relapsing and remitting MS and ~45 % in clinically isolated syndromes. More effective treatments such as immunosuppressants or monoclonal antibodies have been associated with long term risks of severe side effects in particular related to the interference with whole subpopulations of lymphocytes and subsequent disruption of the mechanisms of immunosurveillance [27–29]. Therefore, the development of new drugs that have neuroprotective and enhanced repair mechanisms without compromising some positive aspects of the immune system such as immunosurveillance mechanisms is currently needed [30]. We have previously identified a novel GrB-inhibitor, serpina3n. It was isolated from mouse Sertoli cells and forms a complex and stable covalent bond with GrB and thereby inhibits the enzymatic activity of the protease [31]. It has been shown that serpina3n reduces the rate of aortic rupture and death in a mouse model of abdominal aortic aneurysm (AAA) by inhibiting GrB-mediated decorin degradation and thereby enhancing collagen remodeling [32]. Moreover, the same group showed that topical administration of serpina3n accelerates tissue repair and would healing in a mouse model of diabetics [33]. This strong inhibition of GrB activity makes serpina3n a potentially novel therapeutic approach for inflammation-mediated neurodegenerative diseases such as MS. Therefore, in this study, we tested the hypothesis that inhibition of GrB with serpina3n prevents inflammatory-mediated neurodegeneration in vitro and in vivo in the animal model of MS, experimental autoimmune encephalomyelitis (EAE).

## Materials and methods

### Culture of human fetal neurons and T cells

The University of Alberta Biomedical Ethics Committee (UABEC) approved the collection of human brain tissue from therapeutic abortions of 15-20 week fetuses, collection of blood samples from healthy volunteer donors and isolation of human peripheral blood mononuclear cells (PBMCs). The donor’s mother provided informed consent in writing before donating the tissue. Blood donors provided informed verbal consent, and their names were registered in a blood donor registry before participating in the study. Human fetal neurons (HFNs) and PBMCs (T cells) were isolated and cultured as previously described [11]. All animal procedures and experiments were approved by the University of Alberta Health Sciences Laboratory Animal Services.

### Killing assay and serpina3n inhibitory effect

T cells activation, using anti-CD3 and anti-CD28, and killing assay pursued our previous protocol [14]. After 3 days, some portions of activated T cells (total PBMCs) were incubated with a granzyme B-inhibitor serpina3n (100 ng/ml) for 1 h prior to co-culturing with HFNs. To assess the killing assay and inhibitory effect of serpina3n, T cells were co-cultured with HFNs in a 1:1 ratio. The control neuronal culture groups were treated with only AIM-V medium or co-cultured with unactivated T cells. The experimental groups were co-cultured with activated T cells, with Jurkat cells, supernatant pre-treated activated T cells, or with serpina3n pre-treated activated T cells, and the co-culture was kept for 24 h.

### Immunocytochemistry and western blotting

Evaluation of immune-stained neurons was performed as previously reported [14]. For serpina3n-inhibition study, some groups of activated T cell cultures were incubated with 100 ng/ml serpina3n for 2 h prior to co-culture. Cleavage of the cytoskeletal protein was evaluated using antibody against alpha-tubulin (α-tubulin) (1:3000) using western blotting as previously described [14].

### EAE induction and assessment

We induced EAE by subcutaneous immunization with 50 μg of myelin oligodendrocyte glycoprotein 35–55 (MOG_35-55_) in 10- to 12-week-old female C57BL/6 mice (Charles River). The MOG_35-55_ was emulsified in complete Freund’s adjuvant (CFA) (1 mg/ml to a final concentration of 0.5 mg/ml CFA) (Sigma–Aldrich, Oakville, ON). An intraperitoneal injection of 300 ng/mouse Pertussis toxin (Sigma–Aldrich, Oakville, ON) was administered at the time of EAE induction and again 48 h later. Control mice were treated with CFA (0.5 mg/ml) and Pertussis toxin alone and assessed following the procedures described in previous reports [34, 35]. Briefly, after EAE induction, mice were monitored daily, and the clinical signs of EAE were graded on the following scale: Grade 0, normal mouse; Grade 1, flaccid tail; Grade 2, mild hindlimb weakness with quick righting reflex; Grade 3, severe hindlimb weakness with slow righting reflex; Grade 4, hindlimb paralysis in one hindlimb or both.

### serpina3n treatments

Treatment of serpina3n followed different concentrations at different time points. Cohorts of mice were treated with 25 μg serpina3n either at day 7 post EAE induction or at the disease onset of each animal. Other groups of mice were treated with 50 μg serpina3n at both days 7 and 20 post EAE induction. serpina3n was injected via tail vein in a volume of 200 μl using 26G1/2 needle and 1 ml syringe (BD). The EAE experiment was repeated for four times using *N* of 7–10 mice per each group. A person blinded to the experimental groups performed administration of serpina3n.

### Immunohistochemistry and quantification of histology

After 36 days of evaluation, the mice were sacrificed and tissues were processed as previously described [36]. Briefly, the following primary antibodies, rat anti-CD4, rat anti-CD8 (both 1:200, Serotec), and a mouse anti-SMI32/SMI312 (1:500, Wako), were used. The number of CD4^+^ T cells infiltrating the CNS parenchyma within the lumbar portion of the spinal cord was counted in sections of both control and serpina3n-treated groups. To assess axonal damage, SMI32-positive axons were quantified in a similar fashion. A cocktail of SMI32/312 antibody staining was used to assess and quantify axonal/neuronal death.

An observer blind to the specific experimental conditions of the tissue being analyzed carried out all image analyses. In all the above assessments, three sections per slide, two slides per animal, and *n* = 5 animals per group were analyzed.

### Myelin staining

To measure the status of demyelination of the tissues, sections/slides were hydrated in descending alcohol concentrations. After washing in water for 1 min, slides were stained in Eriochrome cyanin (EC) for 1 h. Following EC staining, slides were differentiated in 0.5 % aqueous NH_4_OH for 10 s and then coversliped using Permount (Sigma).

### Statistical analysis

Results were statistically analyzed using GraphPad Prism 5 and presented as mean ± SEM. The groups were compared using ANOVA followed by the Tukey post hoc test for normally distributed data. A two-tail unpaired *t*-test was applied to compare two groups with normally distributed data. *P* values of <0.05 were considered significant. Asterisks represent *P < 0.05 and **P < 0.01.

## Results

### serpina3n attenuates activated T cell-mediated neuronal death

**Fig. 1:**
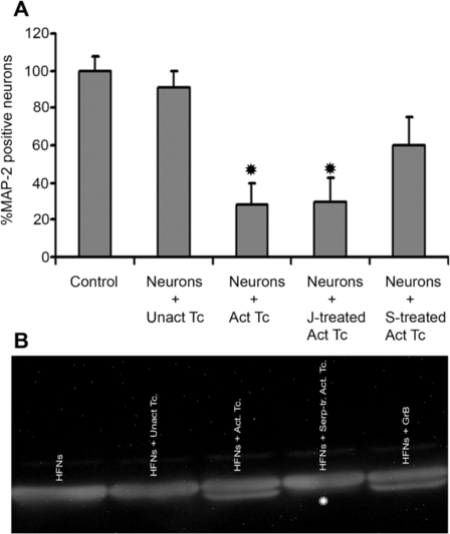
serpina3n attenuates activated T cell-mediated neuronal killing and alpha-tubulin cleavage. **a** Human fetal neurons were treated with T cell media (control) or co-cultured with unactivated T cells, with activated T cells, with Jurkat cells-derived supernatant-treated activated T cells or with serpina3n-treated activated T cells. The culture was immuno-stained using anti-MAP2 antibody. The control group was taken as 100 %. serpina3n treatment significantly reduced T cell-mediated neuronal killing (*P* < 0.05; data is pooled from at least three different donors of neurons and T cells). **b** Western blotting showing that the cytoskeletal protein, alpha-tubulin, remained intact in the control group (neurons treated with media only), and in the groups in which neurons were co-cultured with unactivated T cells. Alpha-tubulin was cleaved in the neurons that were either treated with GrB (positive control) or co-cultured with activated T cells. serpina3n treatment prevented activated T cell-mediated cleavage of alpha-tubulin (indicated by _*_)

We have previously shown that activated T cells induce human neuronal death in vitro [11, 14] via the release of the serine protease granzyme B [14]. Both CD4^+^ and CD8^+^ T cells express GrB and induce neurotoxicity [13, 14]. We also reported that GrB and serpina3n form a complex interaction to the extent that the enzyme loses its activity [31]. Hence, with the objective of preventing T cell-mediated neuronal death, activated T cells (total PBMCs) were pre-incubated with 100 ng/ml serpina3n for 1 h prior to the co-culture with HFNs. Because serpina3n was expressed in Jurkat cells [31], another control group of activated T cells was pre-treated with supernatants collected from non-serpin-expressing Jurkat cells. The positive control was represented by neurons co-cultured with activated T cells whereas the negative controls were neurons co-cultured with either unactivated T cells or media only. Quantification of MAP-2 immuno-reactive neurons revealed that serpina3n treatment significantly reduced activated T cell-mediated neuronal death whereas the supernatant from control Jurkat cells not expressing serpina3n did not show any neuroprotective effect. Average neuronal survival in the groups co-cultured with activated T cells or activated T cells pre-treated with Jurkat cell-derived supernatant was about 30 %. On the contrary, serpina3n treatment increased the neuronal survival to about 60 % (Fig 1a; *P* < 0.05).

We have also previously shown that activated T cells or GrB induce neuronal death by disrupting cytoskeletal proteins such as alpha-tubulin, which is a primary substrate for GrB in neurons [14]. Here, we assessed whether serpina3n treatment prevents the cleavage of this cytoskeletal protein. Western blotting showed that alpha-tubulin was cleaved in the neurons co-cultured with activated T cells or treated with GrB. On the contrary, the cleavage of alpha-tubulin was absent in the neurons co-cultured with serpina3n pre-treated activated T cells. Neurons in the negative controls were treated with media only or co-cultured with unactivated T cells, and no alpha-tubulin cleavage was seen (Fig 1b).

### serpina3n reduces the severity of the disease in EAE

Considering that serpina3n showed to be neuroprotective in vitro, we assessed the effect of serpina3n in vivo in an animal model of MS, EAE. At day 7 post EAE induction, mice were treated with a dose of 25 μg of serpina3n and monitored until day 36. Day 7 was chosen considering that, after EAE induction, it takes about a week for CNS inflammation. Another group of mice received 25 μg serpina3n only when each mouse began to show clinical signs of the disease. Treatment of the mice with 25 μg serpina3n showed a slight trend toward delaying the initiation as well as minimizing the severity of the disease. However, the overall clinical score (measured as sum of scores-SOS) did not show any variation between treated and untreated animals (data not shown).

**Fig. 2:**
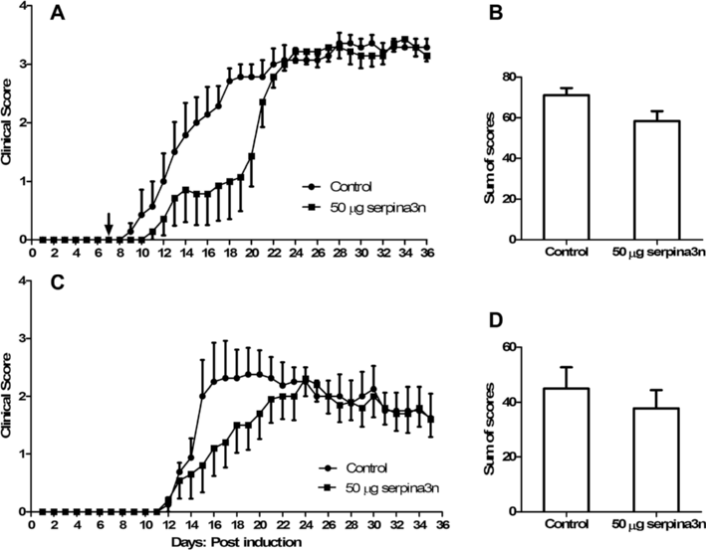
serpina3n delays the onset and attenuates the severity of the disease in EAE. **a** Fifty μg of serpina3n was given at day 7 post EAE induction, and a clinical evaluation followed for 36 days. Mice treated with serpina3n started to show EAE symptoms later than the control group; the severity of the disease was lower in the treated group when compared to the untreated control group. **b** The *graph* shows SOS of “A”. There is no significant difference between the treated and untreated group because the effect of serpina3n was lost after day 20 post EAE induction. **c** Fifty μg of serpina3n was administered IV to the entire experimental group at once when the first mouse in the group showed EAE symptoms. The effect of serpina3n was again lost after day 20/21. **d** There is no difference in SOS in “C” between the treated and untreated group

**Fig. 3:**
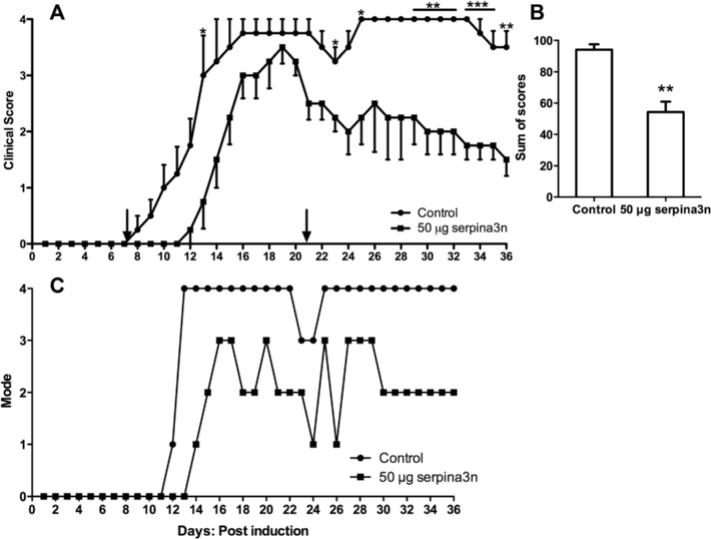
Two injections of 50 μg serpina3n significantly delay the onset and minimize the severity of the disease in EAE. **a** This representative *graph* shows that 50 μg of serpina3n was administered IV at day 7 post EAE induction and delayed the onset of the disease in the treated group. A second injection with the same dose of serpina3n was injected at day 20 and maintained the decreased severity of the disease. **b** Two injections of 50 μg of serpina3n significantly reduced the SOS in the treated group compared to the untreated control (*P* < 0.05). **c** This *graph* shows the modal distribution of the clinical scores. Most of the untreated control mice were severely sick at Grade 4, whereas most of the serpina3n-treated mice were below Grade 3 (*P* < 0.01)

Because of the slight trend we observed with the dose of 25 μg serpina3n, we decided to double the dose to 50 μg of serpina3n. The injection of 50 μg serpina3n at day 7 post-induction delayed the onset of EAE and remarkably reduced the severity of the disease until about day 20 of the course of the experiment. However, this effect was not sustained after day 20, and the clinical scores of untreated and treated mice started to overlap so that the SOS was not different (Fig 2a, b). Similarly, another group of mice received 50 μg serpina3n at the onset of the symptoms. Because of the variability of the onset of the disease, all mice received the treatment at the same time when the first mouse in that group began to show clinical signs of EAE. Treatment with serpina3n at the onset of the disease showed a similar trend to the treatment at day 7 post-induction. serpina3n treatment reduced the severity of the disease, but the effect of the drug was lost almost 14 days post administration (Fig 2c, d). This indicated that serpina3n was losing its effectiveness after 14 days of IV administration and suggested that sequential dosing on days 7 and 20 may be beneficial. This double administration was compared, in the same experiment, to a single injection at day 7 post-induction. In this case, injection of 50 μg serpina3n at day 7 significantly delayed the onset and severity of the disease. Because of the worsening of clinical symptoms around day 20, a second injection with an equal dose was performed at day 20. With this second administration, the severity of the disease was significantly decreased when compared to the control group. This was shown by a significant difference in SOS between the untreated and treated mice (Fig 3a, b; *P* < 0.05). Interestingly, modal analysis revealed that almost all of the untreated mice were severely sick at Grade 4 whereas most of the serpina3n-treated mice were below Grade 3 (Fig 3c; *P* < 0.01).

### serpina3n reduces axonal injury but does not block infiltration of CD4^+^ and CD8^+^ T cells into the CNS

**Fig. 4:**
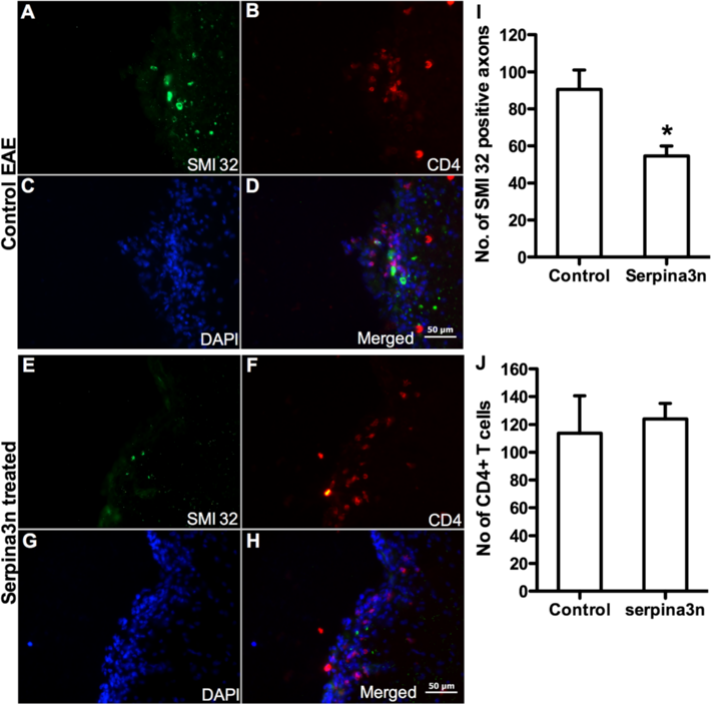
serpina3n significantly reduces axonal injury without interfering in the infiltration of CD4^+^ and CD8^+^ T cells to the CNS. Non-phosphorylated SMI32 positive axons in tissue sections from **a** untreated control group, **e** serpina3n treated group. **b** Number of infiltrating CD4^+^ T cells into the lumbar sections of untreated control mice and **f** Infiltrating CD4^+^ T cells in serpina3n-treated mice. **c** and **g** DAPI-stained cells in untreated control and serpina3n-treated groups, respectively. **d** Merged micrograph from panels **a**, **b**, and **c. h** Merged micrograph from panels **e**, **f**, and **g**. (Scale bars, 50 μm). **i** Quantification of SMI32-positive axons in the control and serpina3n-treated groups (*P* < 0.05). **j** Quantification of infiltrating CD4^+^ T cells in the control and serpina3n-treated groups

To assess whether the improvement of neurological disability in the serpina3n-treated mice was due to a reduction of axonal injury, immuno-staining was conducted using non-phosphorylated anti-SMI32 antibody. Quantification of SMI32-positive axons in the contra-lateral columns (see Additional file 1: Figure S1) in the lumbar section of the spinal cord revealed that 50 μg serpina3n significantly reduced (by about 50 %) the number of injured axons in the treated mice compared to control animals (Fig 4a, e, i; *P* < 0.05). The effect of serpina3n on the transmigration and subsequent infiltration of inflammatory cells through the blood brain barrier is unknown. Interestingly, quantification of CD4^+^ T cells in the lumbar section of the spinal cord revealed that the number of infiltrating CD4^+^ T cells was equal in both control and serpina3n-treated mice (Fig 4b, f, j). The infiltrating T cells were in close apposition or proximity with the injured axons (Fig 4d, h). The same was true for CD8^+^ T cells; however, the number of infiltrating CD8^+^ T cells was low in both groups (data not shown). We have previously shown that CD4^+^ and CD8^+^ T cells express GrB and induce neuronal injury and death [13, 14]. CD4^+^ T cell activation is also recently implicated in the progression of MS [37].

### serpina3n reduces axonal injury

**Fig. 5:**
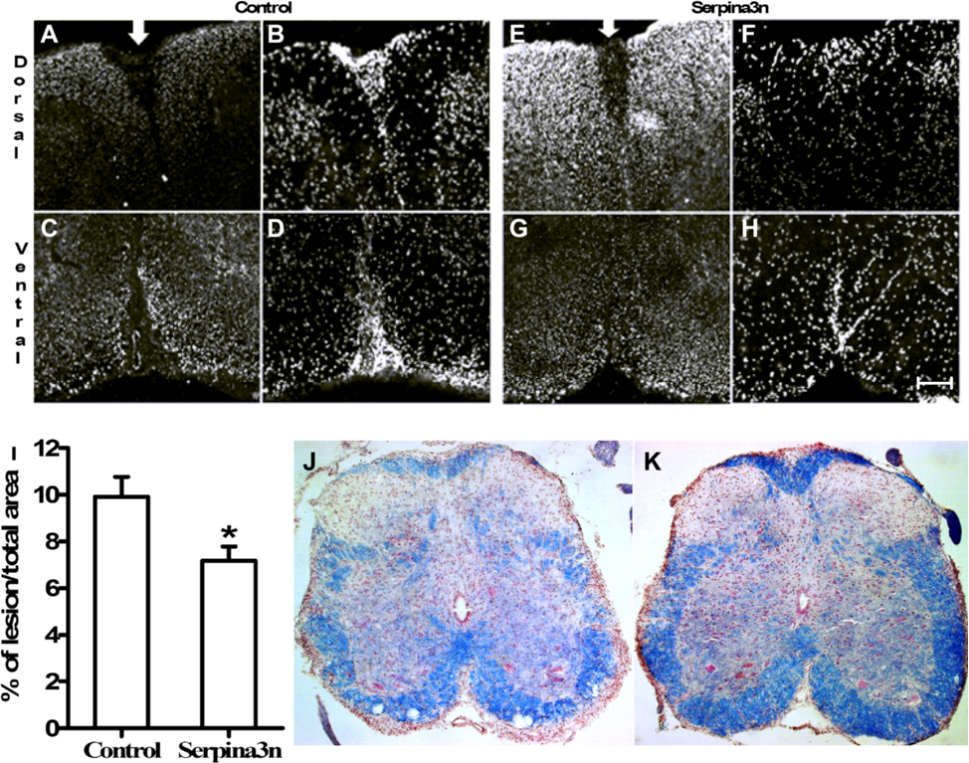
serpina3n significantly reduces inflammatory cell-mediated axonal death. In the untreated control group, representative micrographs showing SMI32/312 immuno-reactive (**a** and **c**) and DAPI-stained (**b** and **d**) sections in the lumbar cord of the dorsal (**a** and **b**) and ventral columns (**c** and **d**). In the serpina3n-treated group, representative micrographs showing SMI32/312 immuno-reactive (**e** and **g**) and DAPI-stained (**f** and **h**) sections in the lumbar cord of the dorsal (**e** and **f**) and ventral columns (**g** and **h**). *Arrows* indicate the lesion area or “black hole”. (Scale bars, 50 μm). **i** This graph shows that the percentage of lesion area/total area of the columns is significantly bigger in the untreated control group when compared to serpina3n-treated group (*P* < 0.05). **j** and **k** serpina3n reduces the magnitude of demyelination. Eriochrome cyanin-stained representative sections showing the scale of demyelination in untreated control (**j**) and serpina3n-treated (**k**) groups, respectively

To assess the amount of axonal loss in the spinal cord, we measured the areas within the white matter without axons, also described as “black holes” as previously shown [34]. We used a cocktail of antibodies that recognize both the phosphorylated and non-phosphorylated forms of neurofilaments to detect healthy and damaged axons in the spinal cord. Sections were immuno-stained with a cocktail of SMI32/312 antibodies. SMI32 stains a dephosphorylated form of 200-kdDa neurofilament subunit (NF200) that appears in injured axons, whereas SMI312 recognizes the phosphorylated NF200 occurring in healthy axons. Areas with no signals to the cocktail of antibodies or lacking both the dephosphorylated and phosphorylated NF200 subunit were used as markers of axonal loss. Therefore, the percentage of the unstained lesion area or black hole in relation to the total area of the column was calculated in the dorsal and ventral columns of the spinal cord. The lesion area (see arrow) was significantly larger in the untreated control (Fig. 5a, c, for dorsal and ventral columns, respectively) compared to the serpina3n-treated mice (Fig. 5b, g). The actual values of the lesion areas are 9.9 % ± 1.7 in the control and 7.1 % ± 1.2 in the serpina3n-treated mice (Fig. 5i; *P* < 0.05). Moreover, DAPI counter-staining revealed that the lesion areas were filled with abundant cells most likely infiltrating inflammatory cells (Fig. 5b, d, f, h).

Myelin staining of the lower lumbar section of the spinal cord with eriochrome cyanin revealed that the integrity of myelin was severely compromised in the control-untreated mice (Fig. 5j) when compared to the serpina3n-treated group (Fig. 5k), indicating the potential role of serpina3n in preventing inflammation-mediated demyelination.

## Discussion

Extensive efforts are currently ongoing to develop new therapeutic strategies for MS that interfere with the underlying causes and mechanisms of neurodegeneration [38]. In this study, we have assessed the neuroprotective effect of GrB-inhibitor serpina3n both in vitro in the human system and in vivo in an animal model of MS, EAE. serpina3n is a member of the superfamily of serpins (serine protease inhibitors), and we previously found that it was the main protective factor in the Sertoli cell-conditioned media and a specific inhibitor of GrB [31]. Others also showed that gonadectomy significantly decreases the expression of serpina3n [39].

GrB is the main cytotoxic weapon released by T-lymphocytes upon activation or encounter with foreign antigens in the periphery. It is well documented that inflammatory cells or cytotoxic T-lymphocytes induce neuronal death [11, 14, 40]. Lymphocytes may possess various lytic mechanisms to induce target cell apoptosis [11, 41]; however, the major contributions seem to come from GrB [17]. It has been previously shown that GrB expressing T cells were in close proximity with demyelinated axons in the parenchyma of acute MS lesions [18], as well as with dying neurons in a rat model of spinal cord injury and cerebral ischemia [42, 43]. In addition, GrB deficient cytotoxic T cells fail to induce nuclear disruption in target cells [24] highlighting the relevance of GrB in the induction of neuronal injury in the mouse system [44]. *In vitro,* activated T cells released GrB into the media and induced cytotoxicity in human neurons [45]. We have shown that GrB enters neuronal cells through the mannose-6-phosphate receptor and induces neuronal apoptosis [14]. All of these reports confirm that GrB mediates neuronal injury and likely contributes to neurodegenerative processes. Inhibiting the enzymatic activity of GrB could potentially be a novel therapeutic approach for inflammatory-mediated neurodegenerative diseases of the CNS such as multiple sclerosis.

Neurons are susceptible to activated T cell-induced neurotoxicity in the absence of added antigen (Ag) and independent of MHC class I or II expression [11]. Most infiltrating T-lymphocytes within MS lesions are Ag nonspecific, and T cells induce collateral bystander axonal injury and neuronal death in the CNS [46–48]. We previously reported that activated T cells and/or GrB induce neuronal death by disrupting the cytoskeletal protein alpha-tubulin [14] indicating that alpha-tubulin is a primary substrate for GrB in neurons. This effect was reversed by serpina3n treatment. Pre-treatment of activated T cells with serpina3n significantly reduced antigen-independent T cell-mediated killing of human neurons in vitro. In addition, western blotting showed that serpina3n prevents activated T cell-mediated cleavage of alpha-tubulin in neurons. Indeed, the interaction of serpina3n with GrB leads to the loss of enzymatic activity of the latter [31]. It has been previously shown that the release of GrB within the target cell triggers cellular apoptosis by cleaving a variety of protein substrates [23, 24, 49] including cytoskeletal proteins [14]. Acute MS neuronal injury is characterized by axonal transection and the formation of axonal spheroids, suggestive of a cytoskeletal disruption [50], and in EAE, T cells mediated the disruption of the microtubule network within neurons [51].

Following our in vitro findings, we tested the hypothesis that serpina3n would induce neuroprotection in EAE. Treating EAE mice with a dose of 25 μg of serpina3n via tail vein at day 7 post-induction slightly delayed the onset of the disease. Treated mice showed decreased clinical severity compared to vehicle-treated controls. However, these differences were not significant most likely because of the large amounts of GrB released by the inflammatory cells and the inability to neutralize or antagonize its effects by a single dose of serpina3n. For this reason, we tested a double dose of serpina3n. Indeed, 50 μg serpina3n substantially delayed the onset of the disease and significantly attenuated the severity of the disease when serpina3n was given at both day 7 and 20 of post EAE induction. This dose-response is in line with the recent finding that serpina3n treatment reduces the rate of aortic rupture and death in a mouse model of AAA in a dose-dependent manner. In this model, serpina3n inhibits GrB-mediated degradation of decorin, a proteoglycan that regulates collagen spacing and fibrillogenesis [32]. In our experiments, injection of 50 μg of serpina3n via the tail vein in a single dose at day 7 post-induction led to a delay in the onset of the disease symptoms, but by day 20 post-induction, the clinical scores of the treated group were overlapping to the control. To overcome this possible pharmacokinetic effect, we decided to inject 50 μg serpina3n in two doses at days 7 and 20 post-induction. In this case, we observed not only a delay in disease onset but also a significant attenuation of the clinical scores. This difference between single dose and double dose could be related to pharmacokinetic of serpina3n and more studies are currently ongoing in our laboratories to further define these mechanisms.

Multiple sclerosis is characterized by inflammation, demyelination, axonal/neuronal destruction, and scar/plaque formation [52–54]. These features are commonly represented in EAE [55]. In this study, serpina3n treatment at the dose of 50 μg twice at days 7 and 20 post EAE induction, significantly reduced axonal and neuronal injury, as well as area of the lesion when compared to the vehicle-treated control mice. Areas completely devoid of staining represent regions where significant axonal loss has occurred. These “black holes” in the tissue were filled with a significant number of DAPI-positive nuclei most likely infiltrating inflammatory cells [8, 9, 42, 43]. The infiltrating inflammatory cells were observed in close contact/proximity with the injured axons as shown in previous reports [18, 42, 43]. Delivering a specific T lymphocyte inhibitor is a major interest in therapeutic strategies [56]. Interestingly in our study, serpina3n treatment did not interfere with the infiltration of CD4^+^ and CD8^+^ T cells into the CNS. However, the overall density of DAPI-stained cells seemed decreased in the tissues from the serpina3n-treated mice compared to the control. This suggests that serpina3n might have additional inhibitory impact on the transmigration of other cells such as macrophages and NK cells that could use GrB for this process [57]. Similarly, others have reported that there was no difference in the level of immune cells infiltration between serpina3n-treated and sham-treated groups in a mouse model of AAA [32]. This highlights that, in most cases, serpina3n is specifically targeting GrB without interfering with the entry of some subpopulations of immune cells, particularly CD4+ and CD8+ T-lymphocytes, into the CNS. This unique property, which is absent in most of the disease-modifying treatments currently available in the market to treat MS, offers serpina3n a potential therapeutic advantage. Indeed, completely blocking the entry of immune cells into the CNS or affecting circulating immune cells and interfering with the mechanisms of immunosurveillance might have serious consequences as demonstrated by the cases of lethal infections that ensued treatments with drugs interfering with the amount of immune cells entering to the CNS [27–29]. Although the effect of serpina3n on demyelination was not the primary objective of our study, we observed decreased demyelination in the spinal cord of EAE mice treated with serpina3n. This could suggest a direct or indirect effect of GrB on myelin that would require further investigations.

Altogether, it is widely accepted that inflammation plays significant roles in the pathogenesis of neurodegeneration in diseases such as MS. Among other mechanisms, inflammatory cells release serine proteases such as GrB and induce axonal damage/neuronal death by disrupting various extra-/intracellular proteins including cytoskeletal proteins. The inhibition of the enzymatic activity of GrB represents an appealing target for therapeutic intervention of these conditions. serpina3n is a serine proteinase inhibitor that forms a complex interaction with GrB and prevents GrB-mediated axonal and neuronal injury, as well as demyelination. In our experiments, serpina3n significantly attenuated the severity of the disease in the animal model of MS, EAE, without interfering with the infiltration of CD4^+^ and CD8^+^ T cells into the CNS. Others also showed that serpina3n was upregulated specifically in astrocytes in ischemic stroke and LPS-induced neuroinflammation models [58]. Our in vitro results robustly show that serpina3n acts on GrB; however, it is also reported that serpina3n reduces the expression levels of matrix metalloproteinases such as MMP-3 and MMP-9 in a mouse model of cardiac remodeling [39]. These MMPs are implicated in neuroinflammatory diseases such as MS and its animal model EAE [59–61]. These findings suggest that serpina3n may undergo complex mechanisms to attenuate inflammation-mediated neurodegeneration. Further studies are warranted to explore serpina3n as well as human analogs as potentially novel therapeutic strategies for MS and other inflammatory-mediated neurodegenerative diseases.

## Additional file

Additional file 1: Figure S1. A representative section from the lumbar part of the spinal cord. The regions under the blue-lined rectangular boxes show the areas where CD4^+^ T cells and SMI32-positive axons were quantified and analyzed. (PPTX 6495 kb)

## Abbreviations

AAA: abdominal aortic aneurysm
Ag: antigen
BBB: blood brain barrier
CNS: central nervous system
EAE: experimental autoimmune encephalomyelitis
EC: eriochrome cyanin
GrB: granzyme B
HFNs: human fetal neurons
MMP: matrix metalloproteinase
MS: multiple sclerosis
PBMCs: peripheral blood mononuclear cells
UABEC: University of Alberta Biomedical Ethics Committee
