# Supplementary material for: Granzyme B-inhibitor serpina3n induces neuroprotection in vitro and in vivo
Source: J Neuroinflammation. 2015 Sep 4;12:157. doi: 10.1186/s12974-015-0376-7 (PMC4558826; doi:10.1186/s12974-015-0376-7)
Supplement: Additional file 1: Figure S1. — A representative section from the lumbar part of the spinal cord. The regions under the blue-lined rectangular boxes show the areas where CD4+ T cells and SMI32-positive axons were quantified and analyzed. (PPTX 6495 kb) [file 12974_2015_376_MOESM1_ESM.pptx]

## Slide 1
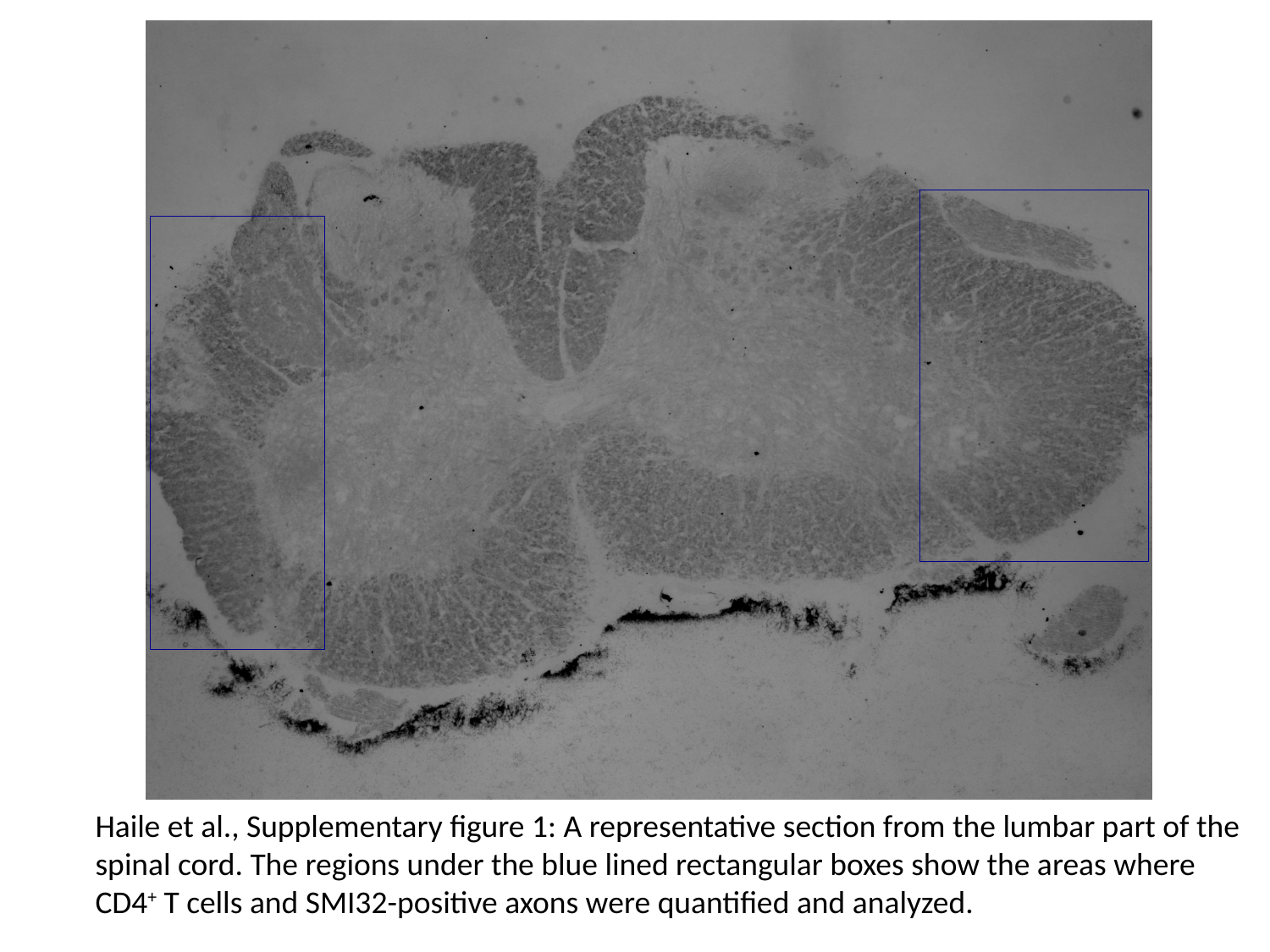

Haile et al., Supplementary figure 1: A representative section from the lumbar part of the
spinal cord. The regions under the blue lined rectangular boxes show the areas where
CD4+ T cells and SMI32-positive axons were quantified and analyzed.
